# Natural history of lung function in spinal muscular atrophy

**DOI:** 10.1186/s13023-020-01367-y

**Published:** 2020-04-10

**Authors:** Camiel A. Wijngaarde, Esther S. Veldhoen, Ruben P. A. van Eijk, Marloes Stam, Louise A. M. Otto, Fay-Lynn Asselman, Roelie M. Wösten-van Asperen, Erik H. J. Hulzebos, Laura P. Verweij-van den Oudenrijn, Bart Bartels, Inge Cuppen, Renske I. Wadman, Leonard H. van den Berg, Cornelis K. van der Ent, W. Ludo van der Pol

**Affiliations:** 1 Department of Neurology, UMC Utrecht Brain Centre, University Medical Centre Utrecht, Utrecht University, Heidelberglaan 100, 3508 GA Utrecht, The Netherlands; 2 Department of Paediatric Intensive Care, University Medical Centre Utrecht, Utrecht University, Utrecht, The Netherlands; 3 Biostatistics & Research Support, Julius Centre for Health Sciences and Primary Care, University Medical Centre Utrecht, Utrecht University, Utrecht, The Netherlands; 4 Child Development and Exercise Centre, Wilhelmina Children’s Hospital, University Medical Centre Utrecht, Utrecht University, Utrecht, The Netherlands; 5 Department of Paediatric Pulmonology, Wilhelmina Children’s Hospital, University Medical Centre Utrecht, Utrecht University, Utrecht, The Netherlands

**Keywords:** Spinal muscular atrophy, Lung function, Natural history

## Abstract

**Background:**

Respiratory muscle weakness is an important feature of spinal muscular atrophy (SMA). Progressive lung function decline is the most important cause of mortality and morbidity in patients. The natural history of lung function in SMA has, however, not been studied in much detail.

**Results:**

We analysed 2098 measurements of lung function from 170 treatment-naïve patients with SMA types 1c–4, aged 4–74 years. All patients are participating in an ongoing population-based prevalence cohort study. We measured Forced Expiratory Volume in 1 s (FEV_1_), Forced Vital Capacity (FVC), and Vital Capacity (VC). Longitudinal patterns of lung function were analysed using linear mixed-effects and non-linear models. Additionally, we also assessed postural effects on results of FEV_1_ and FVC tests. In early-onset SMA types (1c–3a), we observed a progressive decline of lung function at younger ages with relative stabilisation during adulthood. Estimated baseline values were significantly lower in more severely affected patients: %FEV_1_ ranged from 42% in SMA type 1c to 100% in type 3b, %FVC 50 to 109%, and %VC 44 to 96%. Average annual decline rates also differed significantly between SMA types, ranging from − 0.1% to − 1.4% for FEV_1_, − 0.2% to − 1.4% for FVC, and + 0.2% to − 1.7% for VC. In contrast to SMA types 1c–3a, we found normal values for all outcomes in later-onset SMA types 3b and 4 throughout life, although with some exceptions and based on limited available data. Finally, we found no important differences in FVC or FEV_1_ values measured in either sitting or supine position.

**Conclusions:**

Our data illustrate the longitudinal course of lung function in patients with SMA, which is characterised by a progressive decline in childhood and stabilisation in early adulthood. The data do not support an additional benefit of measuring FEV_1_ or FVC in both sitting and supine position. These data may serve as a reference to assess longer-term outcomes in clinical trials.

## Introduction

Spinal muscular atrophy (SMA) is an autosomal recessive neuromuscular disorder (NMD), characterised by a progressive loss of spinal cord motor neurons. This is caused by survival motor neuron (SMN) protein deficiency due to homozygous loss of *SMN1* gene function in all patients [1–3]. SMA demonstrates a remarkably broad range in clinical disease severity, largely explained by variation in the *SMN2* gene copy number [4]. The current classification system distinguishes four SMA types based on age at symptom onset and whether patients acquire the ability to sit or walk independently [3]. The infantile-onset SMA type 1 is the most severe form and characterised by severe muscular weakness, hypotonia, severe morbidity and early mortality due to respiratory failure. Childhood-onset SMA types 2 and 3 are characterised by delayed gross motor development and progressive loss of motor function and muscle strength. SMA type 4 is the mildest type and has an onset in adulthood [1–3, 5].

Increased understanding of the disease course through natural history studies of the past decade has helped clinicians with providing timely supportive care [3, 6] and facilitated clinical trial design to test efficacy of recently developed SMN protein augmenting drugs [7, 8]. Nonetheless, there is still a lack of reference data on several aspects of SMA’s natural history, including lung function, but obtaining additional ‘treatment-naïve’ patient data has become increasingly difficult now that *SMN2*-antisense oligonucleotide treatment is reimbursed in many countries [9].

Reduced lung function is caused by weakness of respiratory muscles and underlies the increased susceptibility to respiratory tract infections. It is the most important cause of morbidity and mortality in patients with SMA [2, 6]. Previous longitudinal studies of lung function included relatively small numbers of SMA patients, did not encompass the entire spectrum of severity or ages, focused on Forced Vital Capacity only, or had limited follow-up [10–15]. Additional natural history data of SMA patients treated according to the standards of care [6], but prior to receiving recently introduced therapies, are important to further improve timing of supportive care and to explore its potential as an outcome measure to evaluate longer-term effects of new treatment strategies [2, 9, 16, 17]*.* To study the natural history of lung function in SMA, we studied outcomes of several commonly used lung function tests (LFTs) longitudinally, using data from treatment-naïve patients participating in a large, population-based prevalence cohort study. We used Forced Expiratory Volume in 1 s (FEV_1_), Forced Vital Capacity (FVC), and Vital Capacity (VC) and here report their longitudinal course across the spectrum of SMA severity.

## Methods

### Design and participants

Patients enrolled in this study are participating in our ongoing prospective population-based prevalence cohort study on SMA in The Netherlands [18, 19]. The study was approved by the local Medical Ethics Committee (No. 09–307/NL29692.041.09) and registered in the Dutch clinical studies and trials registry (https://www.toetsingonline.nl/). Written informed consent was obtained from all participants and/or their parents in case of minors. The reporting of this study conforms to the STROBE statement [20].

For all patients we used multiplex ligation-dependent probe amplification (MLPA; SALSA MLPA kit P021-B1–01, MRC-Holland) to confirm homozygous loss of *SMN1* function and to determine *SMN2* copy numbers. We distinguished SMA types based on age at symptom onset and acquired motor milestones. In case of discrepancies, acquired motor milestones determined final classification. We used previously published additions to also distinguish subtypes (e.g., 2a-b, 3a-b; Table 1) [2, 3, 18, 19, 21]. This is of importance, as a relationship between best acquired motor function and lung function has been reported in several NMDs, including SMA [22]. Patient data were used only if obtained prior to participation in a clinical trial or treatment with SMN protein augmenting drugs (i.e., ‘treatment-naïve’).

**Table 1:** Clinical classification of spinal muscular atrophy

SMA type and sub-classification	Age at onset	Highest achieved motor milestones
1	0–6 months	Never acquires ability to sit unsupported
*0/1a*	*Prenatal / neonatal*	***0/1a****: Symptoms in prenatal and/or neonatal period, no head control*
*1b (‘classic’)*	*1–6 months*	***1b****: No head control and no ability to roll over*
*1c*	*3–6 months*	***1c****: Will usually acquire additional motor skills, such as head control or rolling from supine to prone, or at least to one side at any stage in life.*
2	6–18 months	Able to sit unsupported, not able to walk
*2a*		***2a****: unsupported sitting but not able to stand or walk even with assistance*
*2b*		***2b****: in addition to unsupported sitting also able only with assistance to stand or even walk a few steps*
3	> 18 months	Able to walk unsupported
*3a*	*18–36 months*	
*3b*	*> 36 months*	
4	During adulthood, i.e. ≥ 18 years	Able to walk unsupported

### Lung function tests (LFTs)

We retrieved lung function data from prospectively enrolled patients from two sources. First, we used spirometry data (FEV_1_ and FVC) obtained from patients in our ongoing study [18], using a handheld calibrated spirometer (MicroLab 3500®, PT Medical). These data were obtained prospectively between March 2013 and June 2018, at every study visit. Secondly, we included patients’ (retrospective) spirometry data (Geratherm Spirostik®), collected between July 1991 and July 2018 at the department of pulmonology and Centre of Home Mechanical Ventilation at our hospital [18]. This allowed us to retrieve additional longitudinal FEV_1_ and FVC data, and longitudinal data on VC. All LFTs were measured in sitting position, without corsets or braces.

Additionally, we evaluated the effect of posture by also measuring FEV_1_ and FVC in supine position at every study visit. Normally, measurements in supine position would yield a lower FEV_1_ and FVC [23], but for SMA this was previously assessed only in a small number of patients. We obtained measurements in sitting position first, followed by measurements in supine position after a resting period to prevent a significant influence of fatigability. Lung function tests were performed by a small team of professionals experienced in conducting LFTs in children and adults with NMDs.

All LFTs were measured and reported according to the European Respiratory Society guidelines [24]. We report standardised LFT values, according to the Global Lung Function Initiative [25] and have therefore not transformed data to improve model fitting. Measuring height in SMA patients can be challenging. Arm span was used in most instances as a surrogate measure. In some cases, however, height was used. If so, it was measured preferably in standing position if patients were able to stand, or otherwise in sitting or supine position, using a flexible ruler. The use of a flexible ruler helped avoiding large underestimations due to contractures as much as possible.

### Statistical analysis

We used descriptive statistics to describe baseline characteristics. All available patient data were used for analyses. We assessed longitudinal changes of lung function using linear mixed-effects models (LMMs). We hypothesised a progressive decline of lung function depending on SMA type over time, thus LMMs for the different outcomes contained age (at measurement), SMA type, and an interaction term of these two predictors as fixed factors. Dependency in the data due to repeated measures was accounted for by a random intercept per individual. A random slope for age was added to assess whether there were differences in rates of decline between patients (as measure of disease heterogeneity or between-patient slope variability). We used a likelihood ratio test to evaluate whether the rate of decline over age was significantly different between SMA types. We used estimated baseline values (i.e., the projected intercepts on the y-axes) as a surrogate for lung function outcomes in the earliest stages of life, when these outcomes could not be measured. Model summary statistics and parameters estimates are reported (Table 3).

Because we cannot assume that the natural course of the outcomes of the different lung function tests is completely linear, we also fitted non-linear models. We used smoothed B-spline models with 3 knots, in which polynomial continuous regression lines are computed in between knots [26]. We have provided the visual output of these models to further aid interpretation of the natural history data, as coefficients for such models are not interpretable.

We assessed possible postural influences on FEV_1_ and FVC by comparing repeated LFT measurements of individuals obtained on the same day. As data followed a non-normal distribution (Shapiro-Wilk test *P* < 0.05, non-normally distributed residuals on visual inspection), the Wilcoxon signed-rank test was used. We used *R* (v3.6.0 with RStudio v1.2.1335) for all analyses [27]. The LMMs were fitted using the lmer function of *lme4* (v1.1–21) and *ggplot2* (v3.1.1) was used for data visualisation [28, 29].

## Results

### Demographics

We included 170 patients with SMA types 1c–4, between 4.1 and 73.9 years. Average follow-up was 4.4 years. We were unable to determine *SMN2* copy numbers in two participants (1.2%) due to insufficient quantities of DNA. Baseline characteristics of patients and performed LFTs are shown in Table 2.

**Table 2:** Baseline characteristics and measurements of lung function

**Patients**						
SMA type	Type 1c (*n* = 6)	Type 2a (*n* = 48)	Type 2b (*n* = 34)	Type 3a (*n* = 43)	Type 3b (*n* = 35)	Type 4 (*n* = 4)
M: F	3: 3	19: 29	12: 22	18: 25	18: 17	4: 0
*SMN2* copies						
2	1	1	1	1	1	–
3	5	44	27	21	5	–
4	–	3	5	21	25	4
5	–	–	–	–	3	–
n/a	–	–	1	–	1	–
Mechanical ventilation: *n* (% of total)	5 (83.3%)	23 (47.9%)	3 (8.8%)	5 (11.6%)^b^	1 (2.9%)^b^	0
Median age at start of mechanical ventilation (IQR)	14.6^a^ (13.1–25.9)	12.3^b^ (8.2–16.9)	16.8 (12.7–20.8)	39.9^c^ (35.9–48.3)	40.0^c^	n/a
**Assessments**						
Lung function test		**Patients,***n* (%)	**No. of patient assessments**		**Median no. of assessments per patient** (range)	
FEV_1_		163 (95.9)	784		5 (1–40)	
FVC		167 (98.2)	668		4 (1–32)	
VC		80 (47.1)	646		6 (1–38)	

Legend: *SMA* spinal muscular atrophy; *n* number of patients or assessments; *M* males, *F* females, *SMN2* survival motor neuron 2 gene, *IQR* interquartile range; n/a: not available, *FEV*_*1*_ forced expiratory volume in 1 s, *FVC* forced vital capacity, *VC* vital capacity

^a^: the high median age at which mechanical ventilation was initiated in patients with SMA type 1c is explained by the fact that in The Netherlands it was uncommon to initiate mechanical ventilation for infants with SMA type 1 until recent years, as it was considered not ethical to prolong life without any realistic outlook for further improvements of motor function at a later time. This has changed in the past years, following the introduction of SMN protein augmenting drugs and current clinical drug trials. ^b^: the exact age at which mechanical ventilation was started is unknown for one patient; ^c^: excluded are two patients with SMA type 3a and one patient with type 3b using either bi-level or continuous positive airway pressure for obstructive sleep apnoea syndrome. Ages are shown in years

### Forced expiratory volume in 1 s

**Fig. 1:**
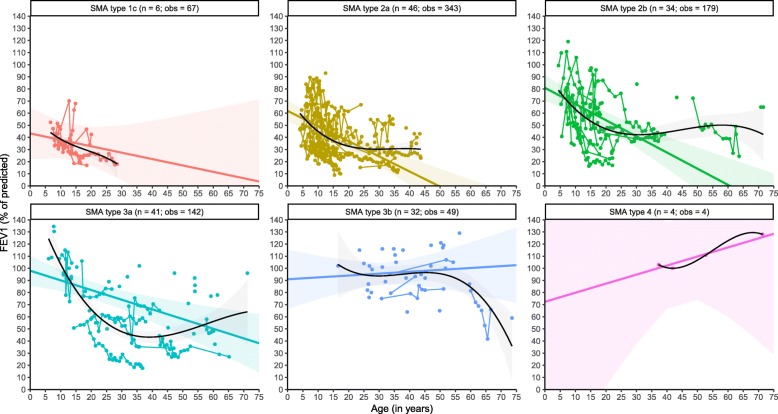
Longitudinal changes of FEV_1_ in SMA. Legend: Linear mixed-model (coloured lines) and non-linear (black) analyses of longitudinal changes in FEV_1_ stratified by SMA type. Solid regression lines indicate the mean values of FEV_1_ and its mean rate of decline over time. Shades represent 95% confidence intervals for the mean rates of decline. n = number of patients; obs = number of observations

We analysed a total of 784 FEV_1_ measurements from 163 patients with SMA types 1c–4 (Table 2). We found a progressive decline of FEV_1_ in SMA types 1c–3a, but not in type 3b. The findings for type 3b likely also extend to type 4, but the limited number of observations precluded reliable estimations. After stratification for SMA type, linear analyses demonstrated significant differences in baseline %FEV_1_ values, i.e., 42% in SMA type 1c, 62% in type 2a, 81% in type 2b, 98% in type 3a, and 100% in type 3b (Fig. 1**,** Table 3). Average annual rates of %FEV_1_ decline differed significantly between SMA types over time (χ^2^(_5_) = 16.381, *P* = 0.0058). There was a decline of 1.29% per year in type 2a, 1.37% in type 2b, and 0.73% in type 3a*.* Due to the limited number of observations, the slope parameters for patients with SMA types 1c (− 0.40%) and 3b (− 0.11%) were not significant (Table 3). Based upon our findings in patients with SMA types 2a and 2b, however, it is likely that FEV_1_ in patients with type 1c will decline, while FEV_1_ values in tube 3b appear to be stable over time and within normal ranges.

**Table 3:** Model parameters

		Fixed Effects				Random effects	
	***n***	Intercept (SE)	95% CI Intercept	Slope	95% CI Slope	Std. dev. Intercept	Std. dev. Slope
FEV_1_							
SMA type 1c	6	42.13 (4.58)	33.89; 50.59	−0.40	−1.42; 0.60 (*n.s.*)	6.11	1.12
SMA type 2a	46	61.71 (4.51)	52.44; 70.60	−1.29	− 1.78; − 0.81	24.35	1.21
SMA type 2b	34	81.37 (6.15)	68.36; 93.53	−1.37	−2.04; − 0.73	25.06	1.18
SMA type 3a	41	97.61 (6.30)	84.80; 110.02	−0.73	−1.11; − 0.35	23.20	0.64
SMA type 3b	32	100.35 (9.17)	81.91; 118.90	−0.11*	− 0.55; 0.32 *(*n.s.*)	16.62	n/a*
FVC							
SMA type 1c	5	49.71 (7.34)	34.65; 68.07	−1.15	−3.29; 0.70 (*n.s.*)	12.60	1.70
SMA type 2a	47	64.20 (5.29)	53.65; 74.64	−1.32	−1.90; −0.76	28.46	1.39
SMA type 2b	34	84.53 (6.07)	71.85; 96.50	−1.40	−2.10; −0.71	23.68	1.28
SMA type 3a	43	96.65 (6.17)	84.08; 108.73	−0.67	−1.06; − 0.31	23.14	0.63
SMA type 3b	34	109.00 (7.42)	94.46; 123.50	−0.23*	−0.58; 0.11* (*n.s.*)	15.35	n/a*
VC							
SMA type 1c	6	44.09 (7.06)	28.41; 60.01	−0.78	−2.35; 0.63 (*n.s.*)	12.84	1.21
SMA type 2a	32	61.01 (4.62)	51.78; 70.16	−1.57	−2.23; −0.94	23.17	1.40
SMA type 2b	22	85.54 (6.98)	69.33; 98.18	−1.65	−2.59; −0.60	25.04	1.46
SMA type 3a	16	96.34 (9.05)	78.17; 114.60	−1.06	−1.71; −0.45	30.07	0.98
SMA type 3b	4	80.99 (19.67)	35.90; 124.82	0.21	−0.77; 1.23 (*n.s.*)	35.12	0.75

Legend: Model parameter estimates, standard errors, and confidence intervals for the linear mixed-effects models are shown. *n*: number of patients in each group

*SE* standard error, *CI* confidence interval, *Std. dev* standard deviation, *n.s.* slope parameter is not significant; *n/a* not available

^*^ due to a too limited number of repeated-measurements ‘age at measurement’ was omitted as a random factor from the mixed-effects model. The slope parameter (i.e. the annual rate of decline in % of predicted) will therefore likely be an overestimation of the true value

Non-linear analyses further confirmed the association of baseline FEV_1_ values and SMA severity, and its progression over time (Fig. 1). The fastest FEV_1_ decline was present at younger ages – exceeding the estimated annual rates of decline from our linear models (Table 3) – followed by a slower further decline during adulthood in SMA types 1c–3a. The available data suggest relatively stable FEV_1_ values over time in types 3b and 4. The limited number of observations of patients with type 3b over 60 years (*n* = 4) likely explains the marked FEV_1_ decline in elderly patients. When stratifying by *SMN2* copy number and SMA type, we found no differences in the longitudinal trajectories for any of the SMA types, with the possible exception of SMA type 3a. Here, patients with type 3a and 4 *SMN2* copies had a slower longitudinal decline in comparison to those with 3 *SMN2* copies.

### Forced vital capacity

**Fig. 2:**
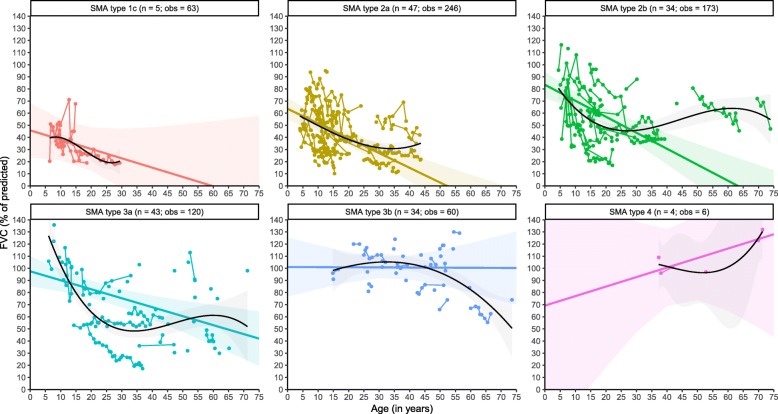
Longitudinal changes of FVC in SMA. Legend: Linear mixed-model (coloured lines) and non-linear (black) analyses of longitudinal changes in FVC stratified by SMA type. Solid regression lines indicate the mean values of FVC and its mean rate of decline over time. Shades represent 95% confidence intervals for the mean rates of decline. n = number of patients; obs = number of observations

In total, we analysed 668 FVC measurements from 167 patients with SMA types 1c–4 (Table 2). Similar to FEV_1_, we observed an FVC decline in the majority of patients over time. After stratification for SMA type, we found a progressive FVC decline in all SMA types, except for type 4 (Fig. 2). At baseline, linear analyses of %FVC demonstrated large differences, i.e., 50% in type 1c, 64% in type 2a, 85% in type 2b, 97% in type 3a, and 109% in type 3b. Significant differences in the average annual rate of decline between SMA types were present (χ^2^(_5_) = 14.202, *P* = 0.014). FVC declined 1.32% per year in type 2a, 1.4% in type 2b, and 0.67% in type 3a. The slope parameters for SMA types 1c (− 1.15%) and 3b (− 0.23%) were not statistically significant (Table 3). The differences in annual average decline between SMA types 2a and 2b, or 3a and 3b were not significant (*P* > 0.05). Due to the limited repeated-measurements for type 3b, the estimated annual decline (− 0.23%) will likely be an overestimation. In fact, the available data indicate relatively stable values over time for type 3b.

Comparable to FEV_1_, non-linear analyses show that FVC decline is most pronounced at younger ages, exceeding the estimated annual rates of decline from our linear analyses. This is followed by a slower rate of decline or even stable course during adulthood in SMA types 1c–3a, whereas FVC remained relatively stable in type 3b throughout life. The steep decline in SMA type 3b from 55 years onwards is likely explained by limited measurements from older patients. The number of observations for patients with type 4 was too small for reliable estimations (Fig. 2).

### Vital capacity

**Fig. 3:**
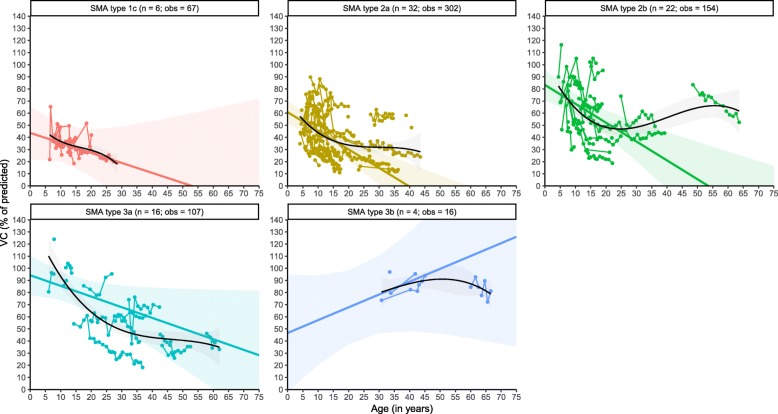
Longitudinal changes of VC in SMA. Legend: Linear mixed-model (coloured lines) and non-linear (black) analyses of longitudinal changes in VC stratified by SMA type. Solid regression lines indicate the mean values of VC and its mean rate of decline over time. Shades represent 95% confidence intervals for the mean rates of decline. n = number of patients; obs = number of observations

Our FVC findings were further supported by a total of 646 VC measurements from 80 patients with SMA types 1c–3b (Table 2). Similar to FVC and FEV_1_, in the majority of patients we observed a VC decline with increasing age. Linear analyses demonstrated large differences in baseline %VC values, i.e., 44% in SMA type 1c, 61% in type 2a, 86% in type 2b, and 96% in type 3a. The predicted average baseline value of 81% for SMA type 3b is likely an underestimation due to the limited number of observations (Fig. 3**,** Table 3). Average rates of yearly VC decline were significantly different between SMA types (χ^2^(_4_) = 10.223, *P* = 0.037) and averaged 1.57% in type 2a, 1.65% in type 2b, and 1.06% in type 3a, whereas the slope parameters were not significant for SMA types 1c (− 0.78%) and 3a (+ 0.21%) due to the limited number of observations for these groups (Table 3). The small difference in slope parameters between patients with types 2a and 2b was not significant (*P* > 0.05). Available data suggest that VC in type 3b was relatively stable over time and within normal ranges. Non-linear analyses further indicate that the longitudinal pattern of VC decline is similar to what we found for FEV_1_ and FVC, i.e., the steepest decline is expected at younger ages in the majority of SMA types, followed by a relatively stable course or slower further decline during adulthood (Fig. 3).

### Postural influence on lung function tests

**Fig. 4:**
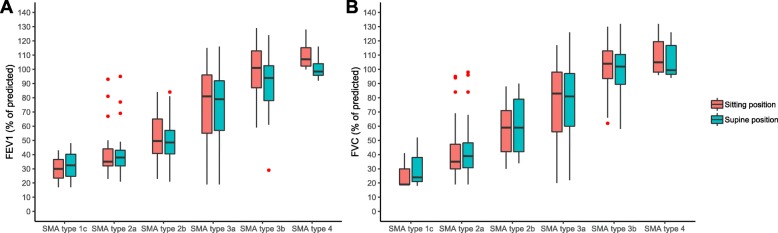
Postural influence on FEV_1_ and FVC measurements. Legend: Comparison of FEV_1_ (**a**) and FVC (**b**) measurements obtained in sitting (red) and supine (blue) position, stratified for SMA type. Small red circles indicate outliers

We assessed postural effects on FEV_1_ and FVC using data from 117 and 162 patients, respectively (Fig. 4). FEV_1_ values differed significantly at group level, although with very small absolute differences: median FEV_1_ was 73% vs. 72%, and mean FEV_1_ was 70.4% vs. 67.9%, respectively (W = 4107, *P* = 0.0101, r = 0.166, sitting vs. supine position). FVC values, however, did not differ significantly: median FVC was 75.0% vs. 77.5% and mean FVC was 72.7% vs. 73.1%, respectively (W = 5485.5, *P* = 0.847, r = 0.01). Differences between FEV_1_ or FVC obtained in sitting vs. supine position were not influenced by disease duration.

## Discussion

Here, we describe the natural history of lung function in treatment-naïve patients with SMA based on a large number of assessments of FEV_1_, FVC, and VC, in a cohort that encompasses the entire spectrum of SMA severity. At baseline, FEV_1_, FVC, and VC are significantly lower in more severe SMA types (1c, 2a), affected to a lesser extent in type 2b and virtually normal in type 3a. Longitudinal decline of lung function in SMA patients is most pronounced during childhood and stabilises in early adulthood. Patients with late-onset SMA (types 3b and 4) are likely to have a stable lung function throughout life, with some exceptions to the rule.

Several relatively small studies previously evaluated the natural history of lung function in patients with SMA [10–14, 30–38]. FVC was studied most frequently and progressive FVC decline has been reported, caused by progressive respiratory muscle failure, limited lung and chest wall growth, and scoliosis progression [39, 40]. Previously reported rates of FVC decline are, however, different from our data. For example, Khirani suggested that patients with SMA type 2 (*n* = 7) had an earlier but comparable rate of decline compared to patients with type 3 (*n* = 9) [13]. Werlauff found no significant difference between patients with SMA type 2 younger and older than 20 years (*n* = 42, cross-sectional data) [33]. By contrast, our findings indicate that rates of decline differ between SMA types 2 and 3, are not constant over time, and may even stabilise in adulthood. Our data also suggest that there are important differences of lung function already at a very young age (i.e., from ‘baseline’ onwards), possibly caused by a more rapid decline in the first years of life, specifically in more severely affected patients. These differences with previous studies are likely explained by the much larger number of observations in our work, facilitating more accurate comparisons between SMA types.

Non-linear FVC analyses showed that the fastest progression is expected during childhood, followed by relative stabilisation during early adulthood in SMA types 1c–3a. This pattern has previously also been noticed by Ioos in a study in which virtually all FVC measurements were obtained before the age of 25 years [37], and more recently by Trucco in a cohort of paediatric patients with SMA types 2 and 3 [15]. For SMA types 3b and 4 there are, to the best of our knowledge, no longitudinal studies available for comparison. Our data suggest that in most of these patients FVC remains relatively stable.

In addition to FVC, we longitudinally analysed FEV_1_ and VC. There are very few previous studies on these outcomes for patients with SMA, impeding meaningful comparisons. Given the large number of observations in our work, we conclude that both FEV_1_ and VC seem to follow a pattern similar to FVC: significant differences are already present at baseline between SMA types, possibly caused by a rapid decline in the first years of life, specifically in more severely affected patients. This is followed by a yearly decline of 0.2–2% during childhood and adolescence, and a relative stable phase during adulthood. At group level, VC and FEV_1_ values remain normal throughout life in SMA type 3b. The available data for patients with SMA type 4 in our work was very limited. However, given the characteristics of SMA type 4 it is likely that these patients will have normal longitudinal values as well. Nonetheless, some individuals with type 3b or 4 may show progressive worsening of lung function that warrants continued awareness. The limited available data on older patients with types 3b and 4 precluded further analyses to identify the characteristics that predict for such a decline.

In our work we stratified patients using the SMA classification system with some modifications that reflect acquired motor milestones other than sitting (type 2) or walking unsupported (type 3). This approach has been helpful in previous studies to uncover differences of the natural history between SMA types. For example, in comparison to type 2a, patients with SMA type 2b are less likely to use mechanical ventilation later in life and require scoliosis surgery at older ages [18, 41]. Here, we have shown that these differences are also present for lung function at baseline. Together, it underscores the prognostic usefulness of additional motor milestones, such as rolling and standing with assistance (Table 1), in addition to sitting and walking unsupported that are used in the current classification system [3, 42, 43].

The general progressive pattern of lung function decline in patients with SMA identified in our work is rather similar to the observed progressive pattern of muscle strength decline in patients with SMA [19], but in adults and particularly those with milder SMA types (i.e., types 3b and 4) lung function may be more stable than skeletal muscle strength. As previously suggested, lung function is therefore a suitable longitudinal outcome measure for patients with SMA, at least until early adulthood [17].

An effect of posture on LFT outcomes has previously been reported for patients with NMDs. Higher outcomes are usually reported for measurements obtained in supine position, possibly due to a mechanical advantage of the diaphragm and muscle fibre stretching [31, 33, 37, 44]. However, we found no significant differences when comparing FVC measured in sitting and supine position. Furthermore, the differences between FEV_1_ measurements were so small that we consider them clinically irrelevant. Given the size of our cohort, measurement standardisation, and consistency across SMA types and patients’ ages, our findings question the usefulness of measurements in both positions, especially as they are time-consuming and relatively difficult to perform in wheelchair-bound patients.

Our work has several strengths. First, we provided baseline and longitudinal reference data not only for FVC, but also for FEV_1_ and VC. Secondly, the large size of our cohort, including a large number of repeated-measurements and relatively long follow-up, allowed for more detailed longitudinal analyses. Finally, LFT therapists and physicians experienced in performing LFTs in paediatric and adult patients with NMDs conducted all tests, assuring measurement reliability.

We also acknowledge several limitations of our work. Broad confidence intervals around both intercept and slope in very young children and elderly patients indicate considerable inter-individual variation. This is partly explained by the inability to reliably perform LFTs in young children and inclusion of a limited number of elderly patients. Secondly, the observed patterns of pulmonary function decline, characterised by relative stabilisation during adulthood, may partially also be explained by the fact that the most severely affected patients could have been lost to follow-up at higher ages, for example due to shorter survival or need for invasive mechanical ventilation. This could particularly be the case for patients with SMA types 1c and 2a. However, we have also observed this pattern in patients with SMA types 2b and 3a, in whom invasive mechanical ventilation is not initiated frequently and survival is not shortened or to such a limited extent that it becomes irrelevant in this context [18, 21, 45, 46]. A similar caution for the interpretation of data of more mildly affected patients applies (i.e., types 3b and 4), as patients with long term stable symptoms could have been lost to follow-up as well.

Our analyses are based upon an uneven distribution of repeated-measures data across SMA types. Most patients with repeated lung function assessments had SMA types 2a, 2b, or 3a. This is caused by the fact that these patients regularly have follow-up visits at the pulmonology department or Centre of Home Mechanical Ventilation at our hospital, because of a higher likelihood of requiring supportive therapy (e.g., cough assistance or mechanical ventilation) in comparison to those with types 3b and 4. The relatively limited number of (repeated) observations for SMA type 1 is explained by the fact that survival in type 1 is short and most of these patients will require (invasive) mechanical ventilation and usually will be lost to follow-up for regular LFTs. Furthermore, we were not able to report LFT results related to known confounders, like severity of (corrected) scoliosis, use of airway clearance techniques, or mechanical ventilation. However, we consider this less important as we focussed on the natural history with treatment according to the standards of care, which include scoliosis correction, airway clearance techniques, and/or mechanical ventilation [3, 6]. LFTs are known to be influenced by respiratory tract infections [17], which were not taken into account. Finally, lung function is not solely defined by the 3 main outcomes used in our work. Data on several other outcomes, including peak cough flow, peak expiratory flow, and maximal inspiratory and expiratory pressures would further improve our understanding of lung function in patients with SMA and should be addressed in future work.

## Conclusions

The natural history of lung function in SMA is characterised by a progressive decline, particularly in SMA types 1c, 2, and 3a. This decline is most pronounced in (early) childhood and stabilises in early adulthood. Our data do not support additional benefits of measuring FEV_1_ or FVC in both sitting and supine position. Our data may serve as a reference to assess longer-term outcomes in clinical trials.

## Data Availability

The model summary statistics and parameters estimates shown in Table 3 allow reproduction of all linear mixed-models. Further data that support the findings of this study are available from the corresponding author, upon reasonable request.

## Abbreviations

95% CI: 95% confidence interval
FEV_1_: Forced expiratory volume in 1 s
FVC: Forced vital capacity
IQR: Interquartile range
LFT: Lung function test
LMM: Linear mixed-effects model
MLPA: Multiplex ligation-dependent probe amplification
NMD: Neuromuscular disease
SMA: Spinal muscular atrophy
SMN: Survival motor neuron
VC: Vital capacity
